# Assessment of Availability and Human Health Risk Posed by Arsenic Contaminated Well Waters from Timis-Bega Area, Romania

**DOI:** 10.1155/2017/3037651

**Published:** 2017-10-15

**Authors:** Marin Senila, Erika Levei, Oana Cadar, Lacrimioara Ramona Senila, Marius Roman, Ferenc Puskas, Mihaela Sima

**Affiliations:** ^1^INCDO-INOE 2000, Research Institute for Analytical Instrumentation, 67 Donath, 400293 Cluj-Napoca, Romania; ^2^Electronic April Cluj-Napoca, 3-5 Pasteur, 400349 Cluj-Napoca, Romania; ^3^Romanian Academy, Institute of Geography, 12 Dimitrie Racovita, 023993 Bucharest, Romania

## Abstract

Mobilization of As from geological materials into ground and drinking water sources may represent an important threat to human health. The objective of this study was to assess the As concentration and availability in underground water used as drinking water sources. Water samples were collected from public and private wells in Timis-Bega area of Pannonian Basin, West Romania. Total-dissolved As measured after “classical” filtration of water samples was in the range of 0.10–168 *μ*g L^−1^, thus exceeding the guideline value in majority of the samples. The aim of this study was also to assess the “truly dissolved” concentrations of As considered as available concentrations, in well waters, after passive sampling by Diffusive Gradients in Thin-films (DGT). The results showed that over 70% of total-dissolved As is in available forms. The obtained data were used to evaluate the risks of using the wells as drinking water source. Hazard quotients for ingestion and dermal pathways and hazard index (HI) for exposure to As were calculated. The HI values > 1 found that majority of samples indicated a health risk for local residents.

## 1. Introduction

Arsenic (As) is a widespread metalloid in the earth's surface which can occur naturally associated with igneous and sedimentary rocks. In addition, anthropogenic activities can discharge high contents of into the environment [1]. As occurs in environment in four oxidation states: arsenate (+5), arsenite (+3), elemental arsenic (0), and arsine (−3) [2]. Elemental arsenic is insoluble in water, but arsenic salts have variable solubility depending on pH [3]. Arsenite is the most abundant As species, while arsenate is stable in oxygenated aquatic environments. In groundwater, As is found mainly in inorganic forms [4], that are considered much more toxic than the organic one [5]. Due to its toxicity, As is ranked on the first position in the list of priority substances compiled by the United States Department of Health and Human Services, Public Health Service [6, 7], and has low regulatory limit (10 *μ*g L^−1^) in drinking water [8]. High As contents may cause adverse health effects on human: dermal and cardiovascular diseases, skin and bladder cancer, diabetes mellitus, and so forth [9, 10]. Thus, As in the drinking water sources is of important concern as it may pose important health risk [11].

Surface water (lakes, rivers) and groundwater (aquifers) are the most common drinking water sources. Drinking water contamination by toxic elements had received keen attention around the world, as As concentrations above the regulatory limit were found all over the world: Bangladesh, India, China, Vietnam, USA, Argentina, Mexico, Chile, Canada, Hungary, Serbia, Croatia, Slovakia, Romania, and so forth [12–14]. Generally, the As-contaminated drinking waters are obtained from aquifers as a result of geothermal sources which influence the As concentration and speciation [15].

The toxicity is influenced both by total concentration and by availability of element [16, 17]. Consequently, environmental regulations should include along the total elements thresholds, the evaluation of risk exposure to toxic elements in terms of their availability. However, the development of reliable and easy to use methods to measure elements availability is necessary. One of the methods that allow the determination of species availability is Diffusive Gradients in Thin-films (DGT) technique based on Fick's first law of diffusion [18]. If the transport of labile element species to an exchange resin is performed exclusively by free diffusion through a diffusive gel layer of known thickness, the concentration in the bulk solution can be calculated from the element mass measured in the resin [19]. DGT technique uses a simple device that contains a layer of resin impregnated in a hydrogel which accumulate the ions of analyte, overlaid by a diffusive layer of hydrogel through which labile species pass according to their diffusion coefficients [18]. The species measured by DGT depends on the thickness of material diffusion layer, the effective pore sizes of the diffusion gel, and the type of resin gel. The DGT measure free ions, inorganic complexes, and some organic element complexes, while species found in colloidal and particulate matter are excluded by the diffusive gel (DGT Research). The resin gel accumulates the analyte species according to ions charge: Chelex based resins accumulate cations while Fe-oxide or Zr-oxide gels accumulate anions [10].

The DGT method was used for speciation and preconcentration of dissolved inorganic As in river water [20] and to monitor bioavailable labile metal fraction in a drinking water treatment plant [21]. However, to date, to the best of our knowledge, there is no literature data regarding the use of DGT for As availability assessment in underground drinking water.

The Pannonian Basin, Eastern Europe, covering parts of Hungary, Romania, Serbia, Slovakia and Croatia is recognized by its high content of As in aquifers used as drinking water sources [14, 22]. Consequently, population living in this area, including West Romania, is exposed to high levels of As through drinking waters [23]. In Romania, there are numerous studies on surface waters contamination with metals [24, 25]; however, only a few papers reported the drinking water contamination with As and its effects on human health [14, 23]. Moreover, in the study area, the analysis of As concentration in drinking waters and the risk assessment for human health was not carried out so far.

The aim of this study was to assess the total-dissolved (*C*_TD_) and available (*C*_DGT_) concentrations of As in underground water collected from wells used as drinking water sources in Timis-Bega area, West of Romania, and to evaluate the risks of population exposure to these concentrations. For the first time, DGT-labile concentrations of As was used to assess the risk through dermal exposure of population. To the best of our knowledge, to date, there is no data on the bioavailable As fractions assessment in well waters using DGT.

## 2. Materials and Methods

### 2.1. Sampling

**Figure 1:**
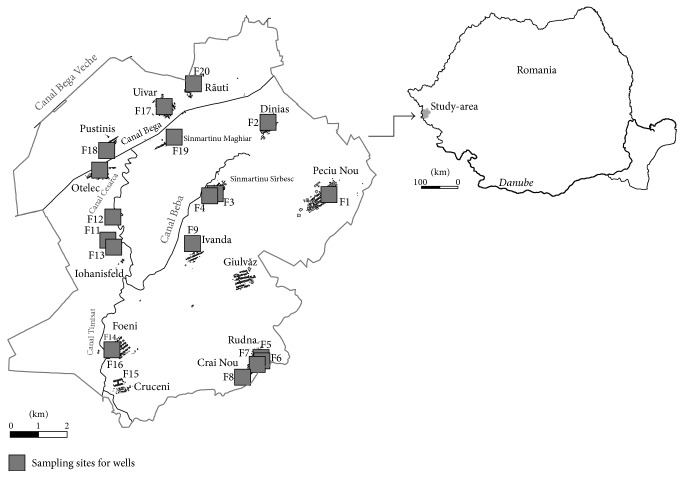
Location of the study area in Romania and the sampling sites.

The study area located in Timis-Bega area, in Banat Plain, part of the Pannonian Basin, West Romania (Figure 1), consists of 13 rural localities grouped in 5 territorial structures and covers 455 km^2^ with 13800 inhabitants. About 40% of households use private- and about 10% public-water wells as the primary source of drinking water. Only 30% of households are connected to the public-water supply system. Wells are used also for agricultural purposes, especially for irrigation of vegetable gardens. The phreatic layer of Timisoara is at a depth, which ranges from 0.5 to 4 m. The depth water layers are more and more numerous from North to the South, from 4 to 9 m, up to 80 m in depth, and contain fresh water, thus providing water for a significant part of the requirements necessary to the urban consumption. The lowest altitude is about 75 m, south of Giulvaz locality.

Water samples were collected from 20 households and public wells (F1–F20, Figure 1). On site, 100 ml of water was filtered through 0.45 *μ*m cellulose acetate membrane filters and acidified with HNO_3_ 65% to pH < 2 for the determination of total-dissolved (*C*_TD_) As. As *C*_TD_ may contain both dissolved and colloidal As forms, to measure really dissolved As, considered to be the available As fraction, the DGT method was used. Three DGT devices were deployed for 24 h in each sampling point. Water temperature at the DGT devices after deployment and retrieval was registered and average value was used to calculate As diffusion coefficient through diffusive gel. The samples were stored in ice box and shipped to laboratory on the same day. In the laboratory, samples were kept refrigerated (4°C) and analysed no later than 1 week from sampling. The pH and electrical conductivity (EC) were determined on site using a portable Multi 340i analyser (WTW, Germany).

### 2.2. Analytical Methods

All reagents used were of analytical grade. Ultrapure water from a Millipore (Molsheim, France) system was used for dilution. Ultrapure HNO_3_ 65% (m/m) (Merck, Germany) was used for acidification and digestion of water samples prior to As determination. The calibration standards were prepared from the 1000 *μ*g ml^−1^ multielement stock solution (Merck, Germany) by appropriate dilutions. The DGT devices were purchased from DGT Research (Lancaster, UK) and contain Fe-oxide, hydrogel, and membrane filter layers. After deployment for 24 h, the DGT devices were retrieved and carefully washed with ultrapure water. As was eluted from the chelating resins with 1 mL HNO_3_ 1 M for 24 h, the eluents were diluted 5 times with ultrapure water before As determination.

The mass (*M*) of As accumulated in the resin gel was obtained using the following [26]:

(1) $$\begin{array}{c} M=\frac{C\times F\times \left(V_{acid}+V_{gel}\right)}{f_{e}}, \end{array}$$

where *C* is the As concentration in eluent, *F* is the dilution factor (5), *V*_acid_ is the volume of eluent (1 mL HNO_3_), *V*_gel_ is the volume of resin gel (0.15 mL), and *f*_*e*_ is the elution factor (0.8).

The concentration of metal measured by DGT (*C*_DGT_) was calculated by the following:

(2) $$\begin{array}{c} C_{DGT}=\frac{M\times \Delta g}{D\times t\times A}, \end{array}$$

where Δ*g* is the diffusion layer thickness (0.078 cm) + membrane filter (0.014 cm), *D* is the diffusion coefficient of As in the resin gel, *t* is the deployment time (approx. 86400 sec), and *A* is the area of the sampling window of the DGT device (3.14 cm^2^) [26].

### 2.3. Instrumentation

The As concentrations were measured by using inductively coupled plasma mass spectrometer ICP-MS ELAN DRC II (Perkin-Elmer, Toronto, Canada) equipped with a reaction cell for reducing the interferences. The pH and electrical conductivity (EC) were determined on site using a portable Multi 340i analyser (WTW, Germany). All the determinations were carried out in triplicate.

### 2.4. Quality Assurance

The accuracy of the analytical results was assured by analysing blank samples, parallel samples, and certified reference material (CRM). Simulated fresh water CRM (SRM 1643e, LGC, UK) was used for the quality assurance. Percent recovery (%) of As in CRM was calculated using the average of five replicates and the relative standard deviation at a 0.05 significance level and was 95.4 ± 6.8%.

## 3. Results and Discussions

### 3.1. General Characteristics of Wells Water

The pH and EC values for underground water samples were in the range of 6.9–7.6 and 0.60–1.25 mS cm^−1^, respectively. The pH of groundwater samples was within the permissible level of drinking water EU Directive [8].

### 3.2. Total-Dissolved Concentrations of As

*C*_TD_ As concentrations in analysed water samples (Table 1) ranged within 0.100–168 *μ*g L^−1^ exceeding the guideline value of 10 *μ*g L^−1^ in 75% of samples. *C*_TD_ were below MAC only in 5 samples (F12 and F13 in Iohanisfeld and F14, F15, and F16 in Foeni), while in the other samples they were much higher (32.4 and 168 *μ*g L^−1^).

The populations that frequently use well waters F1 (Peciu Nou); F2 (Dinias); F3 and F4 (Sanmartinu Sarbesc); F5, F6, and F7 (Rudna); F8 (Crai Nou); F9 (Ivanda); F10 (Otelec); F11 (Iohanisfeld); F17 (Uiuvar); F18 (Pustinis); F19 (Sanmartinu Maghiar); and F20 (Rauti) for drinking, cooking, and personal hygiene are exposed to As concentrations higher than those recommended by regulatory limit.

The found As concentrations were in the same order of magnitude with those reported in previous studies conducted in Pannonian Basin (<0.5–240 *μ*g L^−1^), Bihor and Arad Counties, Romania (0–176 *μ*g L^−1^), Hungary (0–220 *μ*g L^−1^), and Serbia (<10–210 *μ*g L^−1^) [14, 23, 27, 28]. The As concentration in aquifers exceeded 10 *μ*g L^−1^ also in other regions of Europe (<0.3–64.2 *μ*g L^−1^) and Asia (6–4330 *μ*g L^−1^) [5, 29].

### 3.3. Available Concentrations of As

**Figure 2:**
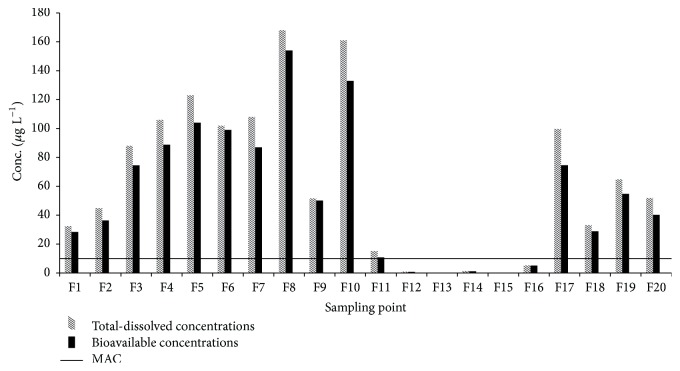
Total-dissolved (pattern bar) and bioavailable (solid bar) concentrations of As in water samples, and the MAC for As in drinking water (horizontal solid line).

*C*_DGT_ and *C*_TD_ are presented in Figure 2. *C*_DGT_ ranged within 70–96% of *C*_TD_. Parts of the As which passed through 0.45 *μ*m membrane filter are colloidal As species and not dissolved As forms and thus are not really bioavailable; however, the percentages of bioavailable contents are quite high. Moreover, for all cases where *C*_TD_ exceeded guideline value, *C*_DGT_ was also above 10 *μ*g L^−1^.

### 3.4. Evaluation of Human Exposure to As through Contaminated Water Use

Drinking water quality is critical for human health and quality of life. The natural and anthropogenic occurrence of As in drinking water has been accepted as a major public health issue. The risk of cancer is estimated quantitatively, while noncancer risk is estimated by using safety factors. In present study, the noncancer risks hazard quotient (HQ) for As were assessed considering ingestion and dermal factors in water, while hazard index (HI) was calculated as the sum of dermal and ingestion HQ. The carcinogenic risk (CR) was determined using reference dose (RfD) and cancer slope factor (CSF) [30].

To calculate HQ, for each village, the mean value of available concentration of As was used.

(3) $$\begin{array}{c} HQ=\frac{ADD}{\text{RfD}}, \end{array}$$

where ADD (mg kg^−1^ day^−1^) represents the average daily dose by ingestion (ADD_ingestion_) and dermal absorption (ADD_dermal_) and RfD is the reference dose for ingestion (RfD_ingestion_ = 0.300 *μ*g kg^−1^ day^−1^) and dermal exposure (RfD_dermal_ = 0.285 *μ*g kg^−1^ day^−1^) [30–33].

(4) ADDdermal=Kp×CDGT×ET×EF×ED×SA×10−3BW×AT×365 days/yr,

where *K*_*p*_ is dermal permeability coefficient (0.001 cm h^−1^), *C*_DGT_ is the available As concentration in water (*μ*g L^−1^), ET is time of exposure (0.5 h day^−1^), EF is the exposure frequency (350 day year^−1^), ED is the exposure duration (30 years), SA is the exposed skin surface (18000 cm^2^), BW is the average body weight (70 kg), and AT is the averaging time (30 years). For noncarcinogenic effects AT = ED. [31–33],

(5) $$\begin{array}{c} ADD_{ingestion}=\frac{C_{DGT}\times IR\times EF\times ED}{BW\times AT\times \left(365\;days/yr\right)}, \end{array}$$

where *C*_DGT_ is the available concentration of As in water (*μ*g L^−1^), IR is the ingestion rate (2 L day^−1^), EF is the exposure frequency (350 days year^−1^), ED is the exposure duration (30 years), BW is the body weight (70 kg), and AT is the averaging time (30 years) [30–32].

To evaluate the total potential noncarcinogenic risk HI was calculated as the sum of HQ_dermal_ and HQ_ingestion_. HQ > 1 or HI > 1 suggests a potential risk for humans or the necessity for supplementary study.  Table 2 presents HQ, HI, and CR for the ingestion and dermal pathways for As in well water for each village. The water from the majority of wells has an As content which poses a risk for human health via ingestion; the highest HI was calculated for well water from Ivanda (15.4132). There are only two villages, Iohanisfeld and Foeni, respectively, where HIs were below 1, indicating no health threat.

The CR was determined using the average daily dose (ADD) and cancer slope factor (CSF). CSF is a measure of chemical potency and is particular to different pollutants. The carcinogenic risk increases linearly as the chemical dose rises. CSF is based on real studies that reflect health effects from carcinogenic pollutants at specific levels [32]. The range of CR acceptable or tolerable by US EPA was 10^−6^ to 10^−4^ [31]. CR was calculated, based on average concentration of As for each village in the study (see (6)) [30]. The US EPA database was used for CSF units [33].

(6) $$\begin{array}{c} CR=ADD\times CSF. \end{array}$$

Except for Foeni, carcinogenic risk of As through ingestion exceeded the tolerable risk 1 × 10^−4^ (Table 2) indicating that the ingestion of water over a long life time could raise the probability of cancer. Ingestion of As at moderate levels may cause dermal and cardiovascular diseases, skin and bladder cancer, and diabetes mellitus, and at high doses As can be even lethal; consequently, special attention should be paid to ensure the safety of local residents.

However, the present approach to evaluate the risks to human health has many uncertainty sources, such as variations in As concentrations and availability in time and location and variations in exposure conditions. Therefore, further investigations are required to evaluate the As concentration and availability in more wells from the area and to corroborate the evaluated risks with the epidemiological data.

## 4. Conclusions

Total-dissolved and available concentrations of As were measured in 20 water samples collected from public and private wells from 13 villages from Timis-Bega area, Romania. The total-dissolved As concentrations in water samples were in the range of 0.1–168 *μ*g L^−1^, thus exceeding the guideline value (10 *μ*g L^−1^) in 75% of the analysed samples. In wells from only two villages, Iohanisfeld and Foeni, As concentrations were below the guideline value. Concentrations measured by DGT technique showed a relatively high availability of As in water samples. Hazard quotients for ingestion and dermal pathways and hazard index for exposure to As were calculated, and HI values > 1 were found in the majority of villages indicating a risk for population's health. For the first time, the DGT-available concentrations of As were used to evaluate dermal exposure to As. Further investigations are necessary for more wells and localities in the area, as well as the information of authorities and residents regarding the risk posed by water consumption.

## Figures and Tables

**Table 1:** Concentration of total-dissolved As in water samples (*μ*g L^−1^, average ± SD, *n* = 3 parallel samples).

Sample	Village	*C*_TD_ As
F1	Peciu Nou	32.4 ± 0.5
F2	Dinias	44.8 ± 0.7
F3	Sanmartinu Sarbesc	88.1 ± 1.8
F4	Sanmartinu Sarbesc	106 ± 3
F5	Rudna	123 ± 2
F6	Rudna	102 ± 3
F7	Rudna	108 ± 3
F8	Crai Nou	168 ± 4
F9	Ivanda	51.6 ± 1.1
F10	Otelec	161 ± 3
F11	Iohanisfeld	15.2 ± 0.6
F12	Iohanisfeld	0.90 ± 0.09
F13	Iohanisfeld	0.21 ± 0.03
F14	Foeni	1.40 ± 0.11
F15	Cruceni (Foeni)	0.10 ± 0.02
F16	Foeni	5.30 ± 0.27
F17	Uiuvar	99.7 ± 2.5
F18	Pustinis	33.1 ± 1.8
F19	Sanmartinu Maghiar	64.8 ± 2.3
F20	Rauti	51.9 ± 1.9

**Table 2:** Hazard quotient for As content in well water in each village from the study.

Village	HQ_ingestion_	HQ_dermal_	HI = ∑HQ_*s*_	CR
Peciu Nou	2.9589	0.0136	2.9725	1.3 × 10^−3^
Dinias	4.0913	0.0189	4.1102	1.8 × 10^−3^
Sanmartinu Sarbesc	8.8584	0.0408	8.8993	3.9 × 10^−3^
Rudna	10.1369	0.0467	10.1837	4.6 × 10^−3^
Crai Nou	4.7123	0.0217	4.7341	2.1 × 10^−3^
Ivanda	15.3424	0.0707	15.4132	6.9 × 10^−3^
Otelec	14.7032	0.0678	14.7710	6.6 × 10^−3^
Iohanisfeld	0.4931	0.0023	0.4954	2.2 × 10^−4^
Foeni	0.2100	0.0010	0.2110	9.4 × 10^−5^
Uiuvar	9.1050	0.0420	9.1470	4.1 × 10^−3^
Pustinis	3.0228	0.0139	3.0368	1.4 × 10^−3^
Sanmartinu Maghiar	5.9178	0.0273	5.9451	2.7 × 10^−3^
Rauti	4.7397	0.0219	4.7616	2.1 × 10^−3^
